# The key role of exudative lesions and their encapsulation: lessons learned from the pathology of human pulmonary tuberculosis

**DOI:** 10.3389/fmicb.2015.00612

**Published:** 2015-06-16

**Authors:** Pere-Joan Cardona

**Affiliations:** Unitat de Tuberculosi Experimental, Fundació Institut d'Investigació en Ciències de la Salut Germans Trias i Pujol, Universitat Autònoma de Barcelona, Centro de Investigación Biomédica en Red de Enfermedades RespiratoriasBadalona, Spain

**Keywords:** tuberculosis, neutrophils, reinfection, encapsulation, interlobular septae, exudative lesions, proliferative lesions, liquefaction

## Abstract

A review of the pathology of human pulmonary TB cases at different stages of evolution in the pre-antibiotic era suggests that neutrophils play an instrumental role in the progression toward active TB. This progression is determined by the type of lesion generated. Thus, exudative lesions, in which neutrophils are the major cell type, are both triggered by and induce local high bacillary load, and tend to enlarge and progress toward liquefaction and cavitation. In contrast, proliferative lesions are triggered by low bacillary loads, mainly comprise epithelioid cells and fibroblasts and tend to fibrose, encapsulate and calcify, thus controlling the infection. Infection of the upper lobes is key to the progression toward active TB for two main reasons, namely poor breathing amplitude, which allows local bacillary accumulation, and the high mechanical stress to which the interlobular septae (which enclose secondary lobes) are submitted, which hampers their ability to encapsulate lesions. Overall, progressing factors can be defined as internal (exudative lesion, local bronchogenous dissemination, coalescence of lesions), with lympho-hematological dissemination playing a very limited role, or external (exogenous reinfection). Abrogating factors include control of the bacillary load and the local encapsulation process, as directed by interlobular septae. The age and extent of disease depend on the quality and speed with which lesions liquefy and disseminate bronchially, the volume of the slough, and the amount and distribution of the sloughing debris dispersed.

## Introduction

In order to be clinically relevant, tuberculosis (TB) lesions must, in general, be radiologically visible. This means a structure with a diameter of not less than 10 mm that can be discerned by an experienced radiologist. It is not easy to achieve such a size in human lungs as powerful local structures, namely the interlobular septae, which enclose secondary lobes, tend to prevent it (Osborne et al., 1983; Webb, 2006). These structures are stimulated by minimal lesions (0.5 mm in diameter) (Medlar, 1955; Lindgren, 1961; Gil et al., 2010), thus showing how difficult it is to overcome this powerful defense. Recent findings concerning the mechanisms that determine the origin of lesions in active TB have led us to take a more in-depth look at pathological data in human pulmonary TB. In particular, renewed interest has been dedicated to the role of neutrophils in the origin of TB. It has been demonstrated that these cells have a relevant role to discern a biosignature for TB in peripheral blood (Berry et al., 2010; Lowe et al., 2012; Bloom et al., 2013). Equally, high concentration of neutrophils has been found in the broncho-alveolar lavage (BAL) of TB patients (Eum et al., 2010). Furthermore, in experimental modeling, neutrophils appear to be instrumental for inducing human-like lesions in mice (Marzo et al., 2014; Vilaplana and Cardona, 2014), as well as in guinea pigs (Ordway et al., 2007), rabbits (Dannenberg, 2006), non-human primates (NHP) (Flynn et al., 2015), goats (Domingo et al., 2009), or cattle (Buddle et al., 2005). These new data reinforce an “easiest” vision of the progression toward TB, based on the instrumental role played by neutrophils, which is less complex and easier to understand than previously hypothesized (Cardona, 2011).

All this evidence suggested the need to review clinical data from the pre-antibiotic era, when a high number of pathological studies from necropsies were performed, in order to try to establish a human model for TB progression.

## The initial phase of infection is silent and unicellular

Once *Mycobacterium tuberculosis* has been phagocytosed by the alveolar macrophages and starts to grow, the initial phase of infection takes place. This phase is silent as it occurs at least 15 days before an initial pre-granuloma appears at the infection site due to the fact that the bacilli grow slowly, duplicating every 24 h until finally necrotizing the cell after around 6 days. Once in the extracellular milieu, they are phagocytosed by neighboring macrophages from the same alveolar sac, probably repeating this cycle once or twice to induce a sufficient inflammatory response to attract the first neutrophils and monocytes (Bru and Cardona, 2010; Cardona and Ivanyi, 2011; Vilaplana and Cardona, 2014).

Evidence for this phase comes from the studies of Wang (1916) and Opie and Aronson (1927), who demonstrated the presence of viable bacilli in samples from healthy parenchyma, a fact subsequently confirmed by Hernandez Pando et al. (2000). Indeed, it has been reported that the time between infection and induction of “primary” TB ranges between 2 and 8 weeks (Wallgren, 1948).

## The quality of the granuloma

Once the infection site has been detected and the immune response triggered, a very important reaction related to the quality of the granuloma generated takes place. There is a widespread consensus that this property is linked to bacillary load and the site at which infection occurs (Pottenger, 1934; Rich, 1944; Canetti, 1955; Medlar, 1955). Thus, the proliferative granuloma, or “tubercle,” is triggered by a low bacillary load and contains epithelioid cells and fibroblasts. This lesion soon progresses to fibrosis and calcification as a result of the encapsulation process induced by interlobular septae, which enclose secondary lobes in humans (Osborne et al., 1983; Webb, 2006), thus meaning that only a very low bacillary load can be detected.

**Figure 1:**
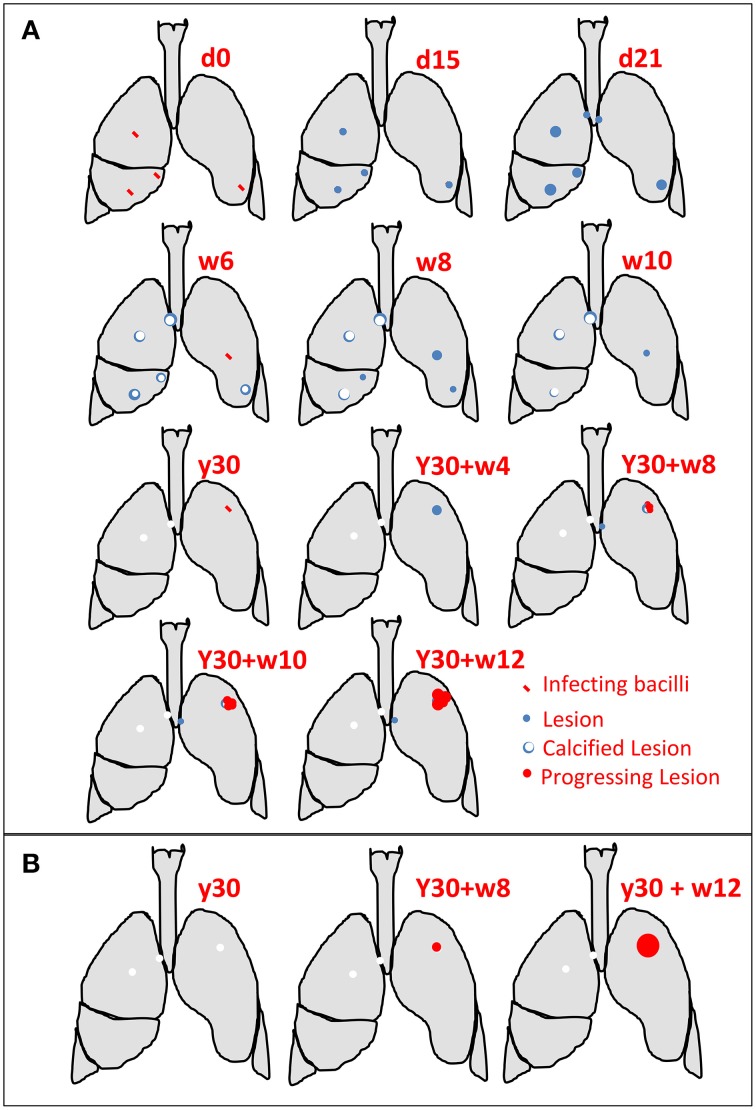
**Examples of progression toward active TB**. **(A)** Different reinfections occurs. These are usually controlled until the upper lobe is infected, with local progression by generation and coalescence of neighboring lesions. **(B)** The classical paradigm in which an old lesion acquired during childhood reactivates and generates TB (d, day; w, week; y, year).

Exudative lesions, or local neutrophilic condensations, are triggered by a high bacillary load. Infection of the upper lobes favors their development and represents a higher probability of generating new lesions as a result of bronchogenic dissemination. Moreover, their high bacillary load also increases the likelihood of necrosis, thus forming a large progressive lesion and favoring liquefaction, sloughing, and cavitation (Pottenger, 1934; Rich, 1944; Pagel and Toussaint, 1948; Canetti, 1955; Medlar, 1955) (Table 1 and Figure 1).

**Table 1:** **Take-home messages**.

**Infection of the upper lobes**		
Progressing	Internal	Exudative lesions
		Bronchogenic local dissemination
		Coalescence of lesions
	External	Exogenous reinfection
Abrogating	Control of bacillary load	
	Encapsulation of the lesions	

The factors that determine the evolution towards TB.

Interestingly, an increase in the percentage of neutrophils in peripheral blood has been used as an indicator for TB progression in the past (Flinn and Flinn, 1930; Pottenger, 1934; Rich, 1944; Kayne and O'Shaughnessy, 1948).

## The upper lobes and tropism for TB

Because of a phenomenon of gravidity, the upper lobes have a higher alveolar pressure and higher diameter than the rest of the lung, thus reducing the capillary density, in addition to having less mobility and thus less lymphatic drainage (Dock, 1954; Glenny and Robertson, 2011). As a result, these lobes are subjected to a lower immunological surveillance due to their lower connectivity with the regional lymph nodes, and therefore have a higher chance of accumulating lesions locally, thereby increasing the bacillary load and resulting in larger lesions. In this regard, a number of different pathological conditions localized in the upper lobes have been reported to be caused by a delayed lymphatic clearance (Gurney and Schroeder, 1988). Moreover, this factor has a limited duration as normal lymphatic drainage is restored every day when the patient rests, an observation that led to prolonged rest being prescribed to TB patients in the past to improve their health status by stopping TB progression (Dock, 1946).

What seems to be important is the markedly lower breathing amplitude compared with the lower lobes (Guo et al., 2011). This may allow an increased local accumulation of bacilli after the destruction of infected macrophages (for at least 2/3 of the day), thus increasing the multiplicity of infection (MOI) of the incoming macrophages and favoring the induction of necrosis (Lee et al., 2006) and the accumulation of neutrophils at the site (Gan et al., 2008). Equally, as a consequence of the increase of pro-inflammatory cytokines, such as IL-6 or IL-8 (Redford et al., 2010; Lowe et al., 2012), and the production of IL-17 by the neutrophils themselves (Khandpur et al., 2013), once the bacilli have been drained to the lymph node and have triggered an immune response, these factors drain with the lymph fluid and help to generate Th17 lymphocytes which, once attracted to the lesion, maintain the infiltration with neutrophils (Lowe et al., 2012).

It has also been found that lesions in the upper lobes exhibit lower calcification (Medlar, 1955). This is surprising considering that the pH of the upper lobes is higher due to the local lower removal of CO_2_, which should favor calcification (Gurney and Schroeder, 1988). As calcification also requires stabilization of the lesion, this is related to a lower encapsulation capacity, which may be due to the lesser ability of the interlobular septae to react against minimal lesions as a result of the enormous stress to which it is subjected (Suki et al., 2013) as the lung must support its own weight. Similar to a suspended coil spring, the largest alveoli and the greatest stress in the lung are found in the apex, and this may weaken the elastic fibers (Gurney and Schroeder, 1988).

Interestingly, a high pH has also been reported to favor the induction of foamy macrophages, as weak bases tend to concentrate intracellularly at higher extracellular pH (Gurney and Schroeder, 1988). It has been extensively demonstrated that foamy macrophages are responsible for the drainage of *M. tuberculosis* out of the lesions, thus playing an important role in bronchogenic dissemination (Cardona, 2009), and they have also been linked to the attraction of neutrophils at the onset of exudative lesions induced in C3HeB/FeJ mice (Marzo et al., 2014).

A definitive proof of the favorable conditions for TB progression in the upper lobes is the observation that the nodules are initially disseminated diffusely throughout the lung in miliary TB, whereas in advanced disease, the foci are larger in the upper lobes (2–3 mm) than in the lower (1 mm) (Auerbach, 1944; Felson, 1952; Gurney and Schroeder, 1988) (Table 1).

However, this is not specific to TB as the upper lobes are also involved in a large number of lung diseases (Ryu and Swensen, 2003; Nemec et al., 2013), including cavitated lesions with different infectious origins (*Klebsiella*, *Pneumocystis*, etc.) (Gadkowski and Stout, 2008), primary cancer (Byers et al., 1984), and metastasis (Yanar et al., 2014), or even the presence of chronic obstructive pulmonary disease (COPD) due to the induction of emphysema (Suki et al., 2013).

## Primary or post-primary lesions?

With the systematic use of chest X-rays to diagnose TB from the end of the Second World War (>1945) (Bynum, 2012), the concept whereby initial infection occurs in childhood but, if controlled, is then detected in adulthood in the form of a calcified nodule in the parenchyma and in the draining hilar lymph node (Ghon Complex) soon appeared (Canetti, 1950). However, adults with TB symptoms tend to develop an infiltration in the upper lobe, with no involvement of the draining lymph node (Adler, 1953; Poppius and Thomander, 1957). These findings led to the leading concept (known as the “unitary concept”) that post-primary disease is a consequence of reactivation of an old lesion generated during the primary infection in childhood, which subsequently leads to the haematogenous dissemination of different lesions until an immune response is generated. This idea was supported by the theory that, once a person is infected and has immunity, he/she cannot be re-infected (Stead, 1967).

The absence of reinfection was refuted by studies from different authors. Thus, for example, Lindgrem et al. demonstrated that BCG vaccination merely curtails the size of the granulomas rather than preventing infection after carefully studying lungs from subjects who died as a result of causes other than TB (Sutherland and Lindgren, 1979). Indeed, lesions of different ages can be seen in the same host, thus supporting the fact that a person can be re-infected several times (Medlar, 1955) (Figure 1). In addition, Medlar demonstrated that involvement of the corresponding lymph node, although minimal, is always detected after performing a careful pathological examination (Medlar, 1948). Similarly, Opie and Aronson (1927) and Canetti (1950) demonstrated that calcified lesions tend to kill the bacilli contained in them, thus reducing the possibility for reactivation with time. In this regard, Canetti strongly supported the fact that TB was mainly based on a process of exogenous reinfections (Canetti, 1950). Furthermore, Pottenger postulated the idea that the greater the number of reinfections the higher the possibility of generating a progressive lesion (Pottenger, 1934). It is well known that persons in contact with an active TB case who are infected and have the highest probability of developing active TB are those who are in contact with the patient for more than 6 h every day, thus meaning that they are infected multiple times and increasing the possibility of infection reaching the upper lobes (20% of total lung volume) and the likelihood of different lesions to progress (Fox et al., 2013) (Figure 1).

The lympho-hematological dissemination mechanism was rebutted by Medlar after a careful study of minimal TB cases as, in general, the lymph nodes generate easily controlled proliferative lesions and as a result of the thrombosis observed in the vessels in necrotic tissue (Medlar, 1948). In fact, looking at the murine model, although there is always a systemic infection (the spleen is always infected), pulmonary progression is always caused by the drainage of infected macrophages out of the lesions, following the bronchial tree (Cardona et al., 2003b). However, lympho-hematological dissemination remains important for inducing extrapulmonary infections in children and immunosuppressed subjects (Pottenger, 1934; Rich, 1944; Kayne and O'Shaughnessy, 1948; Medlar, 1948; Canetti, 1950).

Recently, the use of molecular epidemiology techniques has demonstrated that the radiological pattern of primary TB (lesion in the parenchyma of the lower lobes plus lymph node involvement) is related to the immune status of the host, in this case immunodeficiency status, rather than the timing of the infection (childhood or adulthood). Thus, primary disease, taken to be the absence of signs of previous infection (calcification, etc.), can occur as progressive disease in the upper lobes in immunocompetent subjects, whereas it is more frequent in lower lobes in immunosuppressed subjects. Despite this, the most important sign for immunosuppression was the involvement of lymph nodes (Geng et al., 2005).

## Endogenous bronchial dissemination: a key factor for TB progression

The experience of Medlar after studying over a thousand necropsies, including patients who were followed radiologically from the time at which lesions were finally removed surgically, is of enormous value for understanding disease progression. All these studies (Medlar, 1947a,b,c,d, 1948, 1955) suggest that the small initial lesions which develop in the upper portions of pulmonary lobes often undergo necrosis, liquefaction, and sloughing into the bronchi, thus giving rise to progressive pulmonary disease. However, these lesions can also heal completely by a combination of fibrosis and calcification. If this necrotic lesion has a diameter of 1–2 mm when completely healed, it will remain indefinitely as a calcified or hyalinised focus. Furthermore, the larger the lesion, the lower the likelihood that complete healing will occur.

Even if this lesion liquefies and sloughs, endobronchial dispersion of the slough may be limited to a small pulmonary segment, thus preventing further extension and allowing the possibility of complete healing. In other cases, the disease may progress slowly over a number of years, with some foci undergoing healing and the partial and intermittent sloughing of others resulting in new endobronchial dissemination.

On occasions, the original small foci that have calcified may come into direct contact with large necrotic ones undergoing liquefaction, as also observed by Canetti (1950). Equally, the liquefaction and sloughing of initial foci into the bronchi may lead to a large new focus, and these new lesions may repeat the same process over a short period of time. Thus, it appears that clinical pulmonary TB is always initiated by the liquefaction and sloughing of a tiny necrotic lobular pneumonia lesion that develops in the upper lobes (Figure 1).

The age and extent of the disease is conditioned by the speed with which the secondary lesions undergo necrosis, liquefaction and endobronchial sloughing, the volume of the slough, and by the amount and distribution of the sloughing debris dispersed (Medlar, 1955). In this regard, the slough discharge may go on to form part of internal aerosols and infect other remote regions (Cardona, 2009; Cardona and Ivanyi, 2011).

This phenomenon can also be seen on another scale by considering the fact that bacilli can escape from the granulomas via the foamy macrophages, without the need for liquefaction, as a result of drainage of the alveolar fluid. This results in small-scale bronchial dissemination, which is usually drained toward the stomach, but which (in a low percentage) can result in the formation of aerosols that can reach the upper lobes (Cardona, 2009). This mechanism was demonstrated by Meunier (1898), who used gastric lavage to diagnose TB in children, who do not usually have open lesions. This procedure was subsequently applied systematically from 1927 onwards (Stadnichenko et al., 1940). It can also be clearly seen in experimental modeling in small mammals, where constant bronchogenous dissemination can be detected (Cardona et al., 1999, 2003a,b; Guirado et al., 2008; Cáceres et al., 2009), and in larger mammals, such as mini-pigs (Gil et al., 2010). In the former this phenomenon very often occurs due to the lack of interlobular septae, a structure only present in larger mammals (like humans), which enclose secondary lobes, thereby forming a complex network in connection with the pleura in order to allow respiratory function (Osborne et al., 1983; Webb, 2006; Parent, 2015). This structure allows a quick (in around 10 days) and efficacious encapsulation of minimal lesions, thus curtailing alveolar-bronchial dissemination.

Overall, studies show that progression toward active TB is not homogenous in terms of either the quality of the lesions or the onset of their evolution, thus making progress of the disease unpredictable. Indeed, this varies from case to case as it depends on the balance between the progressing and abrogating factors. But it is important to highlight that progression is highly favored in the upper lobes. This is caused mainly by mechanical reasons, leading to a local accumulation of bacilli, and a lower encapsulation capacity (Medlar, 1955).

## Coalescence of lesions is a signpost for active TB

This mechanism arises as a result of the “conglomeration” of lesions usually exhibited by a cavitated lesion, an aspect previously described by Laennec, who highlighted the usual pattern of a circle of tubercles variously softening and discharging their load of tuberculous matter into the established cavity so that, over time, “continuous excavations are frequently observable” (Laennec, 1826; Bynum, 2012). In fact, what Laennec showed us is that TB is a disease based on the progression of lesions and the attempts of the host to “physically” stop this.

## Softening of lesions has been never demonstrated

Another “mainstream” factor is the concept that old lesions can liquefy. Significant research efforts have been invested in demonstrating this experimentally and have provided some data indicating a possible role of myeloperoxidase in the rabbit model, although there is still no solid proof to support this (Dannenberg, 2006). This concept is yet to be demonstrated in humans. Indeed, it will probably be difficult to do so, especially considering the nature of the fibrosis (collagen), which has been shown not to be “softened” by extremely aggressive inflammatory processes like necrotizing fasciitis (Henningham et al., 2015). In addition, it has been shown that the pleura can prevent the dissemination of cavitated lesions via neighboring lobes (Medlar, 1955).

## What can experimental models offer us?

Experimental models can be classified into those that cannot encapsulate lesions (small/medium mammals) and those which can as they have interlobular septae in their lungs (big mammals) (Plopper and Harkema, 2005; Parent, 2015). Murine models exhibit a kind of immunosuppression-tolerance in a small volume that allows a very slow and controlled progression of the lesions toward total occupation of the lungs but with no symptoms (Cardona, 2010). One important characteristic of mice is their lower percentage of circulating granulocytes (10–25%) compared with humans (50–70%) (Mestas and Hughes, 2004), thus making them potentially less prone to the induction of progressive lesions. Indeed, they usually develop proliferative lesions, and in some cases intragranulomatous necrosis, which is immediately fibrosed. Neutrophilic infiltration is discrete unless C3HeB/FeJ mice (Marzo et al., 2014), or heavily immunosuppressed mice (TNF, IFNγ, CD4, iNOS, IL-12, SCID) (Gil et al., 2006), are studied. Extensive necrosis, which fuels extracellular bacillary growth, can be observed in both cases. It is logical to suppose that initial infiltration with neutrophils starts this progression as a consequence of either the genetic background or marked immunosuppression. Interestingly, in the case of C3HeB/FeJ mice, this also depends on the bacillary dose. Thus, when a low dose aerosol is used, the induction of liquefacted lesions is not predictable and it appears that bronchogenic dissemination must take place and locally synchronize in order to coalesce and induce large liquefacting lesions. In contrast, the inoculation of a relatively large challenge dose (around 10^4^ CFUs) intravenously consistently induces infiltration within around 4 weeks post-infection (Driver et al., 2012; Harper et al., 2012; Vilaplana et al., 2013; Dutta et al., 2014; Marzo et al., 2014). Obviously, although radiologically visible lesions cannot be reproduced in this model on a human-like scale, it can nevertheless give us an idea of the nature of the lesions induced (proliferative vs. exudative). In fact, those groups working on these models have paid particular attention to evaluating control of the bacillary load. But little attention has been paid to the nature of the lesions. In fact, in the majority of models, the lesions are essentially proliferative, showing a slow but constant progression of infiltration due to the lack of an efficient encapsulation process.

Although guinea-pigs (GP) are more reactive and tend to generate both proliferative and exudative lesions, they show an overwhelming implication of the lymphatic system that finally becomes larger and more fibrosed than the lungs, eventually resulting in a quick and overwhelming systemic progression (Basaraba et al., 2006). Medium-sized mammals such as NHP and rabbits also show this constant progression, which in some cases can cause liquefaction of the lesions (Medlar, 1955; Dannenberg, 2006; Flynn et al., 2015).

The fact that they have more volume allows large, radiologically visible lesions to form in these hosts, although the lack of interlobular septae means they have insufficient tools to fight the disease. Interlobular septae are only present in big mammals, such as cattle, goats and pigs, therefore only these hosts will be able to answer the question as to what extent the encapsulation process is relevant or not. In our hands, using the minipig model, we have shown that even lesions smaller than 0.5 mm can be encapsulated in less than 2 weeks. This means that encapsulation is a quite quick process (Gil et al., 2010). The scenario resembles the situation that has been described extensively in humans (Pottenger, 1934; Rich, 1944; Kayne and O'Shaughnessy, 1948; Canetti, 1950; Medlar, 1955), clearly indicating that this is a relevant factor that should be taken into account.

The main drawback of all models is that they are based on a single infection, which is not usually the case in the induction of active TB. As such, it would be important to study the influence of multiple reinfection in large mammals in order to be able to better model the human TB process (Fox et al., 2013; Cardona and Vilaplana, 2014).

## Conclusion: why active TB takes place

The formation of large lesions is essential for the disruption of normal physiology, which is how TB hampers the health of the host. Exudative lesions are those which are able to form quickly enough to overcome the protective mechanism of the interlobular septae, which is the local structure that can stop both the local generation of new lesions and their coalescence into larger lesions. Recently, we have been focusing on the induction of liquefaction as a paradigm for disease induction. However, although this is important by being instrumental to dissemination of the infection in the population, it is in fact a transient feature of the disease. In the end, TB appears as a constant dissemination of new lesions that can coalesce or not, becoming liquefacted or not, but always hamper the host's health by continuous destruction of the lung. This is what has to be stopped. What do we know about this balance between tissue infiltration and encapsulation and stabilization in order to be able try to fix it? Fortunately, we have a lot of experimental models in which we can study the majority of the processes involved in the progression toward TB, except for the encapsulation process. This implies the use of large mammals as they are the only ones with the interlobular septae.

Literature findings also show that it is totally irrelevant whether an infection is primary or not as multiple infections, whether exogenous (especially in high incidence countries) or endogenous (where bronchial dissemination has a special relevance), tend to occur. Moreover, although haematogenous dissemination is important for generating extrapulmonary or miliary TB, it has little or no relevance in the progression toward pulmonary TB.

### Conflict of interest statement

The author declares that the research was conducted in the absence of any commercial or financial relationships that could be construed as a potential conflict of interest.
